# *Camellia japonica* Seed Oil Fermented by *Sporidiobolus pararoseus* Prevents Skin Cellular Photoaging by Inducing Autophagy

**DOI:** 10.3390/life16071145

**Published:** 2026-07-10

**Authors:** Bai Lv, Jiale Meng, Xichao Zhang, Guangtao Li, Hongqi Gao, Yi Jiang, Zhanwei Zhou, Gang Chen

**Affiliations:** 1 School of Pharmacy, Qingdao University, Qingdao 266071, China; bailv430@126.com (B.L.); 18790328527@163.com (J.M.); zhangxichao0201@163.com (X.Z.); 2 Anti-Cellular Senescence Joint Research Laboratory of Forest Cabin, Shanghai Jiao Tong University, Shanghai 201600, China; liguangtao@lqxgroup.com (G.L.); gaohongqi@lqxgroup.com (H.G.); jiangyi@lqxgroup.com (Y.J.); 3 Shanghai Forest Cabin Cosmetics Group Co., Ltd., Shanghai 201600, China; 4 Department of Pharmaceutics, China Pharmaceutical University, Nanjing 210009, China

**Keywords:** skin aging, *Camellia japonica* seed oil, longevity yeast oil, autophagy

## Abstract

*Camellia japonica* L. (Theaceae) seed oil is an essential component for skin protection, attributed to its antioxidant properties and anti-aging effects. In this study, *Camellia japonica* seed oil underwent fermentation with *Sporidiobolus pararoseus* (Fell & Tallman, CGMCC No. 39106, commercial name: Longevity yeast of 1021-year-*Camellia japonica*). The post-fermented oil, designated Longevity Yeast Oil (LYO), was used in the present research. Gas chromatography–mass spectrometry analysis demonstrated that methyl esterification of LYO afforded twelve fatty acid methyl esters. We used an aging model in human epidermal keratinocytes and human skin fibroblasts exposed to ultraviolet B (UVB) to explore the anti-skin aging effects and mechanisms of LYO. The results of the CCK-8 assay demonstrated that LYO exhibited no cytotoxicity but instead displayed potential proliferative activity, indicating its excellent safety profile. Quantitative real-time polymerase chain reaction analyses confirmed that LYO downregulated the expression of autophagy marker p62 and upregulated LC3B, thereby activating the autophagic pathway. Further investigation revealed that LYO protected against UVB-induced apoptosis, promoted the synthesis of collagen and elastin, and upregulated the expression of loricrin, filaggrin, and ceramides, effectively reversing UVB-induced skin cellular aging. Notably, we further revealed that LYO alleviated UVB-induced cutaneous photoaging via activating cellular autophagy pathway. In conclusion, LYO demonstrated biocompatibility and conferred protection to skin cells against photoaging, thereby establishing a theoretical basis for the development of innovative autophagy-targeted anti-aging therapies.

## 1. Introduction

Skin acts as the primary barrier of the body against external environmental insults. In contrast to intrinsic aging (chronological aging), photoaging represents characteristic structural and functional alterations in the skin resulting from repeated ultraviolet B (UVB) exposure [1]. Upon penetration of the sebum film and stratum corneum by UVB radiation, reactive oxygen species (ROS) accumulation and DNA damage are induced in skin cells [2,3,4]. Meanwhile, intracellular signaling cascades lead to the upregulation of matrix metalloproteinases (MMPs) [5,6]. Excessive activation of MMP not only accelerates the degradation of collagen and elastic fibers, but also suppresses collagen biosynthesis, ultimately contributing to typical photoaging phenotypes such as increased wrinkle formation, reduced skin firmness, neovascularization, and abnormal pigmentation [7]. Therefore, developing effective interventions that target photoaging holds promise for improving skin health, slowing the aging process, and reducing the incidence of skin disorders, thereby offering substantial translational potential and significant scientific value.

Autophagy is a catabolic and recycling process essential for maintaining cellular homeostasis and energy balance by degrading damaged organelles, misfolded proteins, and redundant cytoplasmic components [8,9]. During skin photoaging progression, autophagy selectively eliminates UVB-induced ROS, damaged mitochondria, and abnormal lipid accumulations, thereby blocking the cascade amplification of damage signals and mitigating cutaneous oxidative stress [10]. By timely clearing DNA damage-associated proteins and harmful metabolic byproducts, autophagy safeguards the integrity of nuclear and mitochondrial DNA and restrains the onset of apoptotic cell death [11,12]. Moreover, accumulating evidence also indicates that autophagy modulates local cutaneous immune responses and preserves skin barrier integrity by degrading pro-inflammatory cytokines and damage-associated molecular patterns, thus restraining UVB-induced chronic low-grade inflammation [10,13,14].

Chemically synthesized autophagy activators, such as rapamycin, induce autophagy by inhibiting the mTOR signaling pathway and significantly enhance autophagic flux in skin cells, thereby improving collagen synthesis in fibroblasts. However, long-term topical application may lead to adverse effects including skin irritation and suppression of immune cell function [15]. In addition, peptide-based autophagy activators, such as Aquatide^TM^ 5000C (Incospharm, Daejeon, Republic of Korea) [16], activate autophagic pathways in skin cells through targeted binding to autophagy-related proteins, exhibiting high specificity. Nevertheless, their clinical application is largely restricted by high production costs and complex preparation procedures. Therefore, the development of natural product-derived autophagy activators confers distinctive advantages and promising application prospects in anti-photoaging skincare.

The seed oil of *Camellia japonica* L. (Theaceae) contains abundant bioactive components, including oleic acid, vitamin E, and squalene. Owing to its excellent moisturizing and anti-aging properties, the oil can effectively alleviate wrinkle formation and maintain skin youthful status [17,18]. However, the mechanisms underlying the photoprotective effects of camellia oil against skin photoaging remain to be elucidated. Compared with conventional pressing techniques, the co-fermentation of camellia oil with specific microorganisms can markedly enhance oil yield as well as the bioactivity and diversity of functional components, aligning with the current research and development paradigm that emphasizes natural origin, safety, and high efficacy [19]. In this study, we used *Sporidiobolus pararoseus* (Fell & Tallman, CGMCC No. 39106, commercial name: Longevity yeast of 1021-year-*Camellia japonica*), originally isolated from *Camellia japonica* roots, to ferment *Camellia japonica* seed oil. The resultant biotransformed oil is termed Longevity Yeast Oil (LYO). Using human epidermal keratinocytes (HaCaT) and human skin fibroblast (HSF) senescence models exposed to UVB radiation [20,21], we investigated the protective effects of LYO against skin photoaging and its underlying mechanisms, thereby filling these critical research gaps and establishing an experimental foundation for the development of LYO-based formulations.

## 2. Materials and Methods

### 2.1. Materials and Reagents

HiScript^@^ II Q RT Supermix and Cham Q Universal SYBR qpcr Master Mix was purchased from Vazyme Biotech Co., Ltd. (Nanjing, China). Apoptosis Assay Kit was purchased from Uelandy Biotechnology Co., Ltd. (Suzhou, China). Cell Counting Kit-8 was purchased from Taoshu Biotech Co., Ltd. (Shanghai, China). The p62 and LC3B primers were purchased from Tsingke Biotechnology Co., Ltd. (Beijing, China). The p21 antibody was purchased from Proteintech Group, Inc. (Wuhan, China). Collagen Type IV antibody was purchased from Abways Co., Ltd. (Shanghai, China). FoxO3a antibody, SIRT1 antibody, MMP-1 antibody, Collagen Type I antibody, Collagen Type III antibody, and elastin antibody were purchased from Huaan Biotechnology Co., Ltd. (Hangzhou, China). Anti-HRP Goat anti-Rabbit was purchased from Abmart Inc. (Shanghai, China). The Collagen Type VII, Loricrin (LOR), Filaggrin (FLG), and Ceramide (CER) ELISA Kit were purchased from Bioswamp Life Science Lab. (Wuhan, China). The PI3K-III inhibitor 3-Methyladenine (3-MA, A8353) was purchased from APExBIO Technology LLC. (Houston, TX, USA). Fermented *Camellia japonica* seed oil, obtained by fermenting *Camellia japonica* seed oil with *Sporidiobolus pararoseus* (CGMCC No. 39106) and separating the oil fraction, was provided by Shanghai Forest Cabin Cosmetics Group Co., Ltd. (Shanghai China). The UVB lamp fitted with an original 9 W UV-B tube from Philips (Shanghai, China) and the UV radiation power meter used in this study were manufactured by UlanPhotonics Co., Ltd. (Suzhou, China).

### 2.2. Fermentative Preparation of LYO

Shake-flask seed culture: The strain stored at -80 °C was thawed at room temperature, inoculated into YM broth medium, and incubated at 28 ± 2 °C with shaking for 16–24 h until OD_600_ reached 8.0–12.0. The culture was then transferred into fresh YM medium at a 10% inoculum ratio and cultured under the same conditions for secondary activation to obtain the shake-flask seed liquid. Seed tank culture: YM medium was prepared at 5% working volume, sterilized at 121 °C, 0.1 MPa for 30 min, and cooled to 28 °C. The shake-flask seed liquid was fully inoculated into the seed tank. The culture conditions were: temperature 28 ± 2 °C, aeration rate 1 vvm, tank pressure 0.03–0.05 MPa, agitation speed 300 r/min, for 18–24 h. The culture was used as fermentation seed when dissolved oxygen recovered and OD_600_ reached 8.0–12.0. Fermentation culture: The basal fermentation medium consisted of glucose 5 g/L, peptone 20 g/L, yeast extract 20 g/L, KH_2_PO_4_ 3 g/L, K_2_HPO_4_ 5 g/L, (NH_4_)_2_HPO_4_ 3 g/L, NaCl 5 g/L, and MgSO_4_·7H_2_O 1 g/L. The pH was adjusted to 6.0 before sterilization. Camellia seed oil was added at a ratio of 50% (*v*/*v*), mixed well, and sterilized at 121 °C, 0.1 MPa for 30 min. After sterilizing the transfer pipeline, the seed culture was inoculated into the fermenter at 5–10% inoculum ratio. Fermentation conditions were: temperature 28 ± 2 °C, aeration rate 1 vvm, tank pressure 0.03–0.05 MPa, agitation speed 200 r/min, for 18–24 h. The fermentation was terminated when dissolved oxygen recovered and pH stabilized. Product collection: After fermentation, the broth was inactivated with high-temperature steam at 80–95 °C for 20–30 min and allowed to stand for 2–3 h. The upper oil phase was collected as LYO.

### 2.3. Analysis of LYO

The fatty acid composition of LYO was characterized using gas chromatography–mass spectrometry (GC-MS). Samples were weighed into 10 mL centrifuge tubes, mixed with 1.5 mL of 14% BF_3_ in methanol and 50 μL of undecanoic acid (20 mg/mL), and incubated at 65 °C for 30 min. After cooling, 1.5 mL of n-hexane and 5 mL of purified water were added. The mixture was vortexed and centrifuged at 3800 r/min for 5 min. The upper layer was transferred into a 1.5 mL LC vial for analysis. Chromatographic separation was carried out on an HP-5MS capillary column (60 m × 0.25 mm, 0.25 μm). The injector temperature was set at 280 °C, with an injection volume of 1.0 μL and a split ratio of 5:1. High-purity helium was used as the carrier gas at a constant flow rate of 1.5 mL/min. The column temperature program was as follows: the initial temperature was 120 °C and held for 1 min, then increased to 170 °C at 6 °C/min with no hold time, subsequently raised to 215 °C at 2.5 °C/min and maintained for 12 min, afterwards heated to 230 °C at 4 °C/min and kept for 10 min, and finally elevated to 280 °C at 10 °C/min and held for 15 min. For mass spectrometry detection, the ion source temperature was 200 °C, and the quadrupole temperature was 150 °C. The transfer line temperature was 260 °C. Electron impact ionization was performed at 70 eV. The mass scan range was set from *m*/*z* 40 to 550, and the solvent delay time was 4.4 min. Identification of fatty acid derivatives was carried out by searching against the NIST Mass Spectral Library (NIST-08), in comparison with reference fatty acid methyl esters standard spectra and the retention times of individual fatty acid methyl esters. The concentration of each volatile compound was calculated using an internal standard method based on undecanoic acid (20 mg/mL, 50 μL). The experiments were conducted with three replicates.

### 2.4. Cell Culture and UVB Irradiation

HSF and HaCaT cell lines were preserved in our lab. HSF cells were cultured in a proprietary medium supplemented with 10% (*v*/*v*) fetal bovine serum and 1% streptomycin/penicillin. HaCaT cells were cultured in DMEM supplemented with 10% (*v*/*v*) fetal bovine serum and 1% streptomycin/penicillin. All cells were incubated at 37 °C in a humidified atmosphere with 5% CO_2_.

Before conducting in vitro UVB exposure, HSF and HaCaT cells were treated with different concentrations of LYO for 24 h, and then immersed in PBS. LYO was dissolved and prepared in medium containing 0.5% ethanol (*v*/*v*). The total UVB dose was 100 mJ/cm^2^ (311 nm, 9 W/01, Philip, Shanghai, China). After irradiation, the PBS was replaced with a culture medium with or without LYO, and incubated in an incubator for 24 h for further experiments.

### 2.5. Cell Viability Assay

To assess the potential cytotoxicity of LYO against HSF and HaCaT cells, we performed CCK-8 assays using Cell Counting Kit-8 (Taoshu Biotech Co., Ltd, Shanghai, China). Briefly, the wells of 96-well plates were seeded with cells, which were treated with different concentrations of LYO for 24 h. Following this treatment period, CCK-8 reagent was added to the wells, followed by incubation at 37 °C for 2 h. To assess cell viability, optical density (OD) values at 450 nm were determined using a microplate reader.

### 2.6. Annexin V-FITC/PI Apoptosis Assay

An Annexin V-FITC Apoptosis Detection Kit was used to detect the apoptosis rate of HSF and HaCaT cells. Cells were seeded into 6-well plates at a density of 5 × 10^5^ cells per well. After incubating for 24 h, cells were pretreated with LYO, followed by UVB irradiation and LYO intervention as described above. Next, the cells were resuscitated with binding buffer, 10 μL Annexin V-FITC, and 10 μL PI were added for 10~20 min in the dark. Finally, the florescence of the cells was tested by flow cytometry.

### 2.7. Western Blotting Analysis

We initially isolated nuclear and cytoplasmic proteins from HSF and HaCaT cells using a nuclear and cytoplasmic protein extraction kit, the concentrations of which were determined by Nanodrop. Whole-cell proteins were similarly extracted using RIPA buffer. Having quantified the proteins, the nuclear and cytoplasmic proteins were electrophoretically separated on 10% SDS-polyacrylamide gels and subsequently transferred to polyvinylidene difluoride (PVDF) membranes. The primary antibodies used for Western blot analysis include those against p21 (1:1000; Proteintech Group, Inc., Wuhan, China), Collagen Type IV (1:1000; Abways Co., Ltd, Shanghai, China), SIRT1 (1:1000), FoxO3a (1:2000), MMP1 (1:1000), Collagen Type I (1:5000), Collagen Type III (1:1000), elastin (1:1000) and β-actin (1:50,000); all were from Huaan Biotechnology Co., Ltd. (Hangzhou, China). Results were normalized to β-actin. The binding of these primary antibodies was determined using a suitable HRP-conjugated secondary antibody (1:50,000, Abmart, Shanghai, China) at room temperature for 2 h, with an Immobilon Western Chemiluminescent HRP Substrate being used for detection of the protein bands. Densitometric analysis was performed using ImageJ2 (version 2.14.0/1.54g) software.

### 2.8. Real-Time Quantitative PCR (RT-qPCR) Analysis

HSF and HaCaT cells were modeled for photoaging and cultured in medium for 24 h. Total RNA was extracted and purified using Trizol reagent (Gene-Better, Beijing, China) according to the manufacturer’s instructions to measure the mRNA expression. The concentration of the RNA was quantified by the Nano One Drop ^TM^ spectrophotometer (Thermo Fisher Scientific, Waltham, MA, USA). cDNA was synthesized using cDNA synthesis reagents (Rever Tra- Ace^®^ qPCR RT Master Mix with gDNA Remover Code No. FSQ-301, TOYOBO, Osaka, Japan), and RT-qPCR was conducted to quantify the expression of autophagy-related genes, utilizing specific forward and reverse primers, the primer sequences were as follows: p62 forward, 5′-GTG AAC TCC AGT CCC TAC AGA TG-3′; p62 reverse, 5′- GCT CCG ATG TCA TAG TTC TTG GT-3′; LC3B forward, 5′- CGC ACC TTC GAA CAA AGA GTA GA -3′; and LC3B reverse, 5′- AAG CTG CTT CTC ACC CTT GTA TC -3′; GAPDH forward, 5′- CTC CGG GTG ATG CTT TTC CT -3′; and GAPDH reverse, 5′- GCC CAA TAC GAC CAA ATC AGA G -3′. This analysis was carried out on the Quant Studio ^TM^ 5 Real-Time PCR System (Applied Biosystems, Thermo Fisher Scientific, Waltham, MA, USA), facilitating the accurate quantification of gene expression through RT-qPCR techniques. The relative mRNA expression levels were calculated using the ΔΔCt method and normalized to the internal reference GAPDH mRNA level for each sample.

### 2.9. ELISA Analysis

Human Collagen Type VII; (COL VII) concentration, Human CER concentration, Human FLG concentration, and Human LOR concentration were determined by ELISA Kit (Bioswamp Life Science Lab, Wuhan, China). Absorbance at 450 nm was measured using an enzyme plate spectrophotometer.

### 2.10. The Relationship Between Autophagy and Anti-Aging

HSF and HaCaT cells were first treated with 200 μg/mL LYO for 24 h. Subsequently, the cells were exposed to 3-methyladenine (3-MA, 5 mM) for 12 h to inhibit autophagy [22]. Following these treatments, total cell proteins and cell supernatants were collected, and protein expression levels were analyzed by Western blot and ELISA.

### 2.11. Statistical Analysis

All experiments were repeated at least 3 times independently, with data expressed as mean ± SD. Statistical analysis and graphs were conducted using GraphPad Prism version 10.1.2 (GraphPad Software, San Diego, CA, USA). For two-group comparisons, Welch’s t-test (an unpaired t-test with Welch’s correction) was used, which is robust to unequal variances and moderate deviations from normality. For multiple-group comparisons, one-way analysis of variance (one-way ANOVA) was applied, followed by Dunnett’s multiple comparisons test to control the risk of multiple-comparison errors. Statistical differences were presented at ns *p* ≥ 0.05, * *p* < 0.05, ** *p* < 0.01, *** *p* < 0.001.

## 3. Results

### 3.1. GC-MS Analysis of LYO

The chemical composition of LYO was determined by GC-MS analysis. A total of 12 fatty acid methyl esters were formed via methyl esterification of LYO (Table 1 and Figure S1). The top five most abundant components were methyl oleate (71.01%), methyl linoleate (11.95%), methyl palmitate (9.82%), methyl stearate (3.39%), and methyl elaidate (2.71%).

### 3.2. LYO Induces Autophagy in Skin Cells

**Figure 1:**
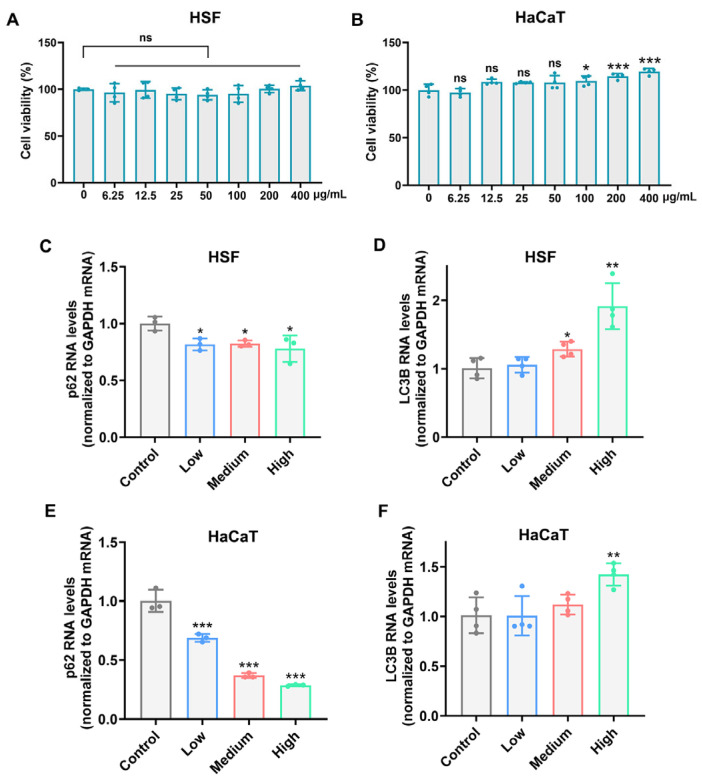
LYO induces autophagy in skin cells. Effect of LYO on CCK-8 level in (**A**) HSF and (**B**) HaCaT cells. (**C**,**D**) The mRNA level of p62 and LC3B in HSF cells after treatments. (**E**,**F**) The mRNA level of p62 and LC3B in HaCaT cells after treatments. Data are presented as mean ± SD (*n* = 4). ^*^ *p* < 0.05, ^**^ *p* < 0.01, ^***^ *p* < 0.001, vs the indicated groups. ns, no significant difference.

Evaluation of cytotoxicity to establish a safe and effective concentration range is an essential prerequisite for determining the research and development value of LYO, the feasibility of mechanistic studies, and its potential for clinical translation. Accordingly, the CCK-8 assay was employed to assess the viability of HaCaT and HSF cells following exposure to varying concentrations of LYO. As shown in Figure 1A,B, LYO exerted no cytotoxic effects on either cell lines. Notably, treatment with 100~400 μg/mL LYO significantly increased viability in HaCaT cells, suggesting that LYO markedly enhances the proliferative capacity of human keratinocytes and thus contributes to the repair and re-establishment of the skin barrier. Subsequently, LYO at concentrations of 50, 100, and 200 μg/mL was selected as the low-, medium-, and high-dose treatment groups, respectively.

Autophagy is a highly conserved intracellular degradative pathway that protects skin cells from UV-induced photoaging damage via multiple approaches, including maintaining cellular homeostasis, suppressing apoptosis, and delaying senescence. As such, it represents a crucial intracellular regulatory mechanism underlying skin anti-aging [10,23]. To investigate the autophagy-inducing effects of LYO on HSF and HaCaT cells, RT-qPCR analyses were conducted to assess the expression of the autophagy markers p62 and LC3B. In HSF cells, the mRNA levels of p62 in the low-, medium-, and high-dose groups were all significantly lower than those in the control group (Figure 1C). The mRNA level of LC3B was significantly upregulated in the medium-dose group, and the upregulation was even more pronounced in the high-dose group, exhibiting an obvious dose-dependent increase (Figure 1D). In HaCaT cells, the mRNA levels of p62 in the low-, medium-, and high-dose groups were all significantly lower than those in the control group (Figure 1E). The mRNA level of LC3B was significantly upregulated in the high-dose group and showed an upward trend in the medium-dose group (Figure 1F). Overall, the expression patterns were consistent with those observed in HSF cells. Microscopic observation showed dose-dependent cytoplasmic vacuolization (red arrows) in HSF and HaCaT cells with increasing treatment concentrations, a characteristic morphological feature of autophagic activation, indicating that the treatment induced cellular autophagy (Figure S2). Collectively, these results indicate that LYO exhibits good biocompatibility with skin cells and can promote autophagy, providing a mechanistic basis for further investigation into its protective effects against UVB-induced skin photoaging.

### 3.3. LYO Alleviates Skin Photoaging Damage Caused by UVB Irradiation

**Figure 2:**
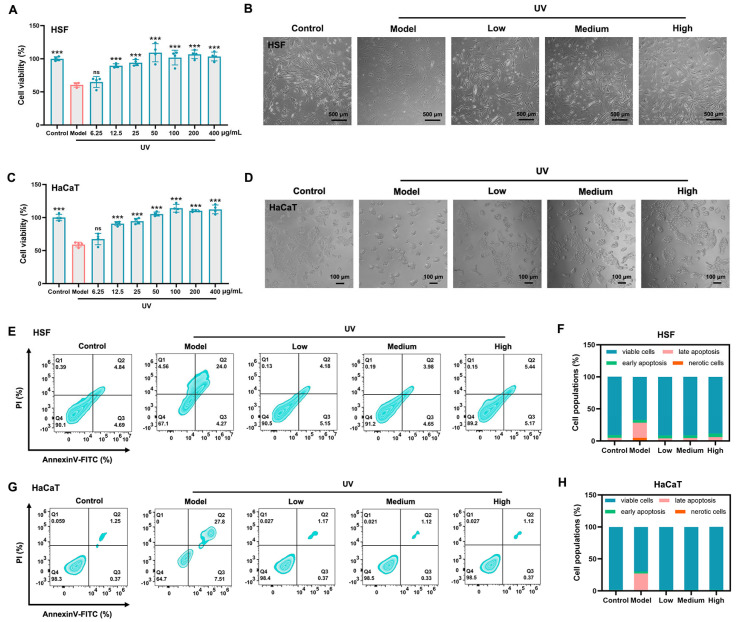
Protective effect of LYO on UVB-induced damage in HSF and HaCaT cells. (**A**) Effect of LYO on the viability of UVB-treated HSF cells determined by CCK-8 assay (n = 4). (**B**) The effect of LYO on the morphology of UVB-treated HSF cells (Scale bar = 500 μm). (**C**) Effect of LYO on the viability of UVB-treated HaCaT cells determined by CCK-8 assay (*n* = 4). (**D**) The effect of LYO on the morphology of UVB-treated HaCaT cells (Scale bar = 100 μm). (**E**) Apoptosis of HSF cells after different treatments analyzed by flow cytometry. (**F**) Quantitative analysis of apoptosis in HSF cells. (**G**) Apoptosis of HaCaT cells after different treatments analyzed by flow cytometry. (**H**) Quantitative analysis of apoptosis in HaCaT cells. The model group denotes the control + UVB group. Data are presented as mean ± SD., *** *p* < 0.001, vs. the indicated groups. ns, no significant difference.

UVB-induced cellular senescence triggers oxidative stress, which in turn leads to inflammatory responses and accelerated aging. Autophagy effectively eliminates oxidative damage caused by UVB irradiation and inhibits apoptosis, thereby exerting a significant photoprotective effect on skin cells [24]. Encouraged by the observation that LYO upregulated autophagy markers, the present study further investigated whether LYO alleviates UVB-induced cellular damage in HSF and HaCaT cells. CCK-8 assay results demonstrated that LYO treatment significantly enhanced cell viability in a dose-dependent manner within the concentration range of 12.5~400 μg/mL (Figure 2A), indicating that LYO markedly improved the survival of UVB-exposed HSF cells. As shown in Figure 2B, the UVB-irradiated group displayed obvious morphological alterations, including shrinkage, cytoplasmic vacuolization, and loss of cell adhesion in HSF cells. Compared with the UVB group, the degree of cellular damage was alleviated and cell density was preserved in the low-, medium-, and high-dose LYO groups, indicating that LYO effectively protects HSF cells against UVB-induced photodamage at the morphological level. Similarly, within the concentration range of 12.5~400 μg/mL, LYO significantly improved the viability of UVB-irradiated HaCaT cells (Figure 2C). HaCaT cells irradiated with UVB exhibited markedly reduced cell density. Compared with the UVB model group, LYO-treated cells showed no obvious damage and displayed morphology similar to normal cells, indicating that LYO protects HaCaT cells against photodamage induced by UVB irradiation (Figure 2D). To further evaluate the photoprotective effect of LYO against UVB-induced injury, flow cytometry was performed to detect apoptosis in HSF and HaCaT cells following UVB irradiation. Compared with the untreated control group, UVB irradiation significantly increased the proportion of early and late apoptotic cells (Figure 2E–H). Quantitative analysis revealed that LYO treatment attenuated this apoptotic response. Collectively, these results demonstrate that pretreatment with LYO attenuates UVB-induced cellular damage in HSF and HaCaT cells.

### 3.4. LYO Mitigates the Progression of UVB-Induced Photoaging in HSF Cells

**Figure 3:**
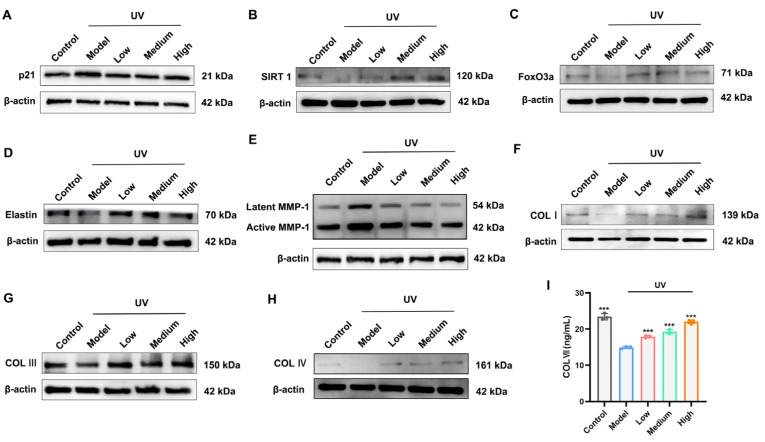
LYO attenuates skin aging in HSF cells. (**A**–**H**) Western blot analysis of the effects of LYO (50 (low), 100 (medium), and 200 (high) μg/mL) on the protein expression levels of p21, SIRT1, FoxO3a, elastin, MMP-1, COL I, COL III, and COL IV in UVB-irradiated HSF cells. (**I**) ELISA analysis of the effects of LYO on COL VII expression in UVB-irradiated HSF cells (*n* = 3). Data are presented as mean ± SD. *** *p* < 0.001, vs. the indicated groups.

p21 is one of the core mediators of cell cycle arrest and a key regulator of cellular senescence [25,26]. As shown in Figure 3A and Figure S3, UVB irradiation markedly elevated the expression of senescence-associated p21 protein in HSF cells compared with the normal control group, indicating that UVB exposure accelerates senescence and cellular damage in HSF cells. Conversely, treatment with LYO at low, medium, and high concentrations reduced p21 protein expression relative to the UVB model group, respectively. Sirtuin 1 (SIRT1) is widely recognized as a novel anti-aging protein implicated in the modulation of cellular senescence and inflammatory responses [27]. In addition, FoxO3a serves as a key regulator of cellular redox homeostasis; by upregulating the transcription of antioxidant enzyme genes, it directly enhances the ability of skin cells to resist oxidative stress induced by UVB irradiation [28,29,30]. As illustrated in Figure 3B–C, UVB exposure downregulated the expression of SIRT1 and FoxO3a in HSF cells, whereas LYO treatment significantly restored their expression levels, suggesting the potential antioxidant activity of LYO (Figures S4 and S5). Elastin, synthesized by dermal fibroblasts, plays an indispensable role in maintaining skin elasticity. As shown in Figure 3D and Figure S6, elastin expression was significantly reduced in the model group compared with the control group. Notably, elastin expression was markedly elevated following LYO treatment.

Upon UVB irradiation, matrix metalloproteinase 1 (MMP-1) exists in both latent zymogen and mature activated isoforms. Latent MMP-1 is converted into catalytically functional, mature active MMP-1 through proteolytic cleavage, which subsequently triggers collagen degradation and ultimately contributes to typical photoaging phenotypes such as wrinkle formation and skin laxity [31,32,33]. Accordingly, the expression levels of MMP-1, collagen I (COL I), collagen III (COL III), collagen IV (COL IV), and collagen VII (COL VII) were determined in the UVB-induced photoaging model of HSF cells. Western blot analysis confirmed that LYO significantly abrogated the UVB-induced upregulation of both latent MMP-1 and active MMP-1 (Figure 3E and Figure S7). Given the role of matrix metalloproteinases in collagen breakdown, LYO treatment effectively restored the levels of COL I, COL III, COL IV, and COL VII, which were diminished by UVB irradiation (Figure 3F–I and Figures S8–10). Collectively, these findings demonstrate that LYO reverses the UVB-induced upregulation of p21 and MMP-1. Moreover, LYO attenuates UVB-mediated skin aging by upregulating the expression of SIRT1, FoxO3a, elastin, COL I, COL III, COL IV, and COL VII.

### 3.5. LYO Mitigates the Progression of UVB-Induced Photoaging in HaCat Cells

**Figure 4:**
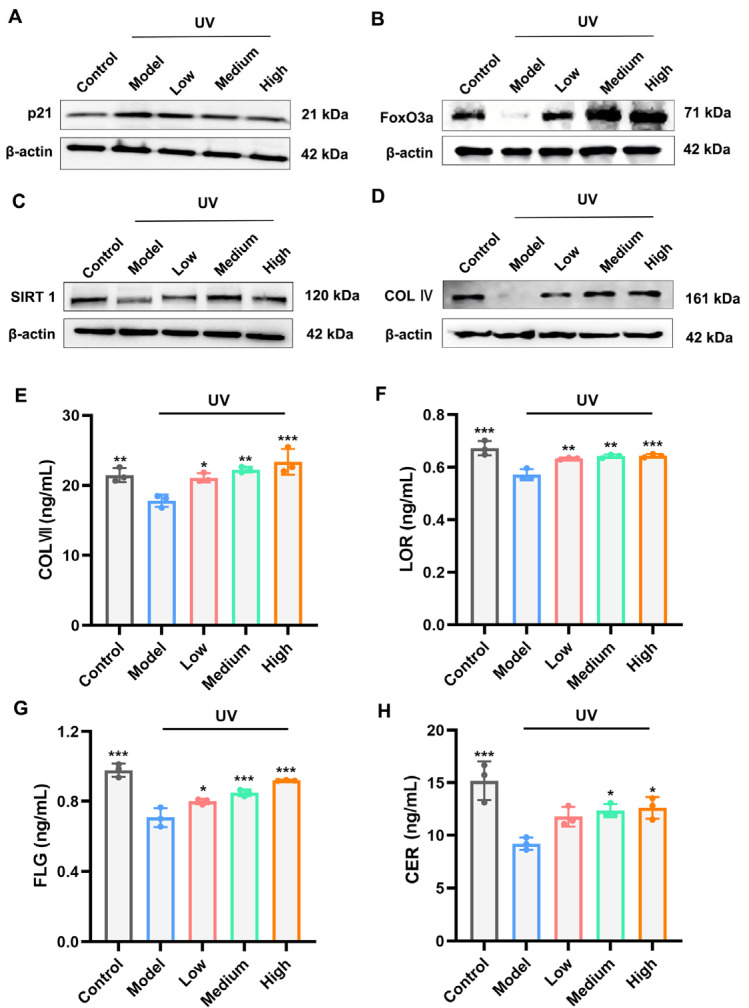
LYO attenuates skin aging in HaCaT cells. (**A**–**D**) Western blot analysis of the effects of LYO (50, 100, and 200 μg/mL) on the protein expression levels of p21, FoxO3a, SIRT1, and COL-IV in UVB-irradiated HaCaT cells. (E-H) ELISA analysis of the effects of LYO on COL VII, LOR, FLG, and CER expression in UVB-irradiated HaCaT cells (*n*= 3). Data are presented as mean ± SD. * *p* < 0.05, ** *p* < 0.01, *** *p* < 0.001, vs. the indicated groups.

Next, we investigated the restorative effects of LYO on UVB-induced senescence in HaCaT cells. As shown by Western blot analysis (Figure 4A–D and Figures S11–14), the expression of senescence-associated regulatory factors was significantly altered in the UVB-treated group compared with the untreated control group. Specifically, the protein level of p21 was upregulated, whereas the expression of FoxO3a, SIRT1, and COL IV was downregulated, indicating that UVB irradiation successfully induced a senescent phenotype and suppressed collagen synthesis in HaCaT cells. Following LYO intervention, the resistance of HaCaT cells to UVB-induced damage was enhanced. Consistent with these observations, the ELISA results for COL VII (Figure 4E) demonstrated that LYO effectively delayed UVB-triggered cellular senescence by promoting collagen synthesis. LOR serves as a structural scaffold of keratinocytes, which strengthens the physical skin barrier, defends against external insults, and alleviates barrier fragility and roughness caused by aging [34,35,36]. FLG contributes to the structural stability of the stratum corneum, improves skin dryness, and attenuates fine lines induced by photoaging [37,38,39,40]. As a key intercellular lipid, CER not only moisturizes the skin and repairs the barrier but also exerts antioxidant effects and reduces collagen degradation [41,42,43]. These three components act synergistically to maintain skin barrier integrity, hydration, and elasticity, thereby delaying the skin aging process. Further quantitative ELISA analysis (Figure 4F–H) revealed that the protein levels of LOR, FLG, and CER expressed by HaCaT cells were significantly elevated in the LYO-treated groups compared with the model group. Moreover, the secretion of these skin barrier- and function-related proteins exhibited a gradual dose-dependent increase with rising LYO concentrations. Collectively, these findings indicate that LYO effectively reverses UVB-induced cellular senescence and barrier dysfunction by upregulating the expression of senescence-associated regulatory factors and skin barrier-related proteins.

### 3.6. Autophagy Activation Contributes to the Anti-Photoaging Activity of LYO

**Figure 5:**
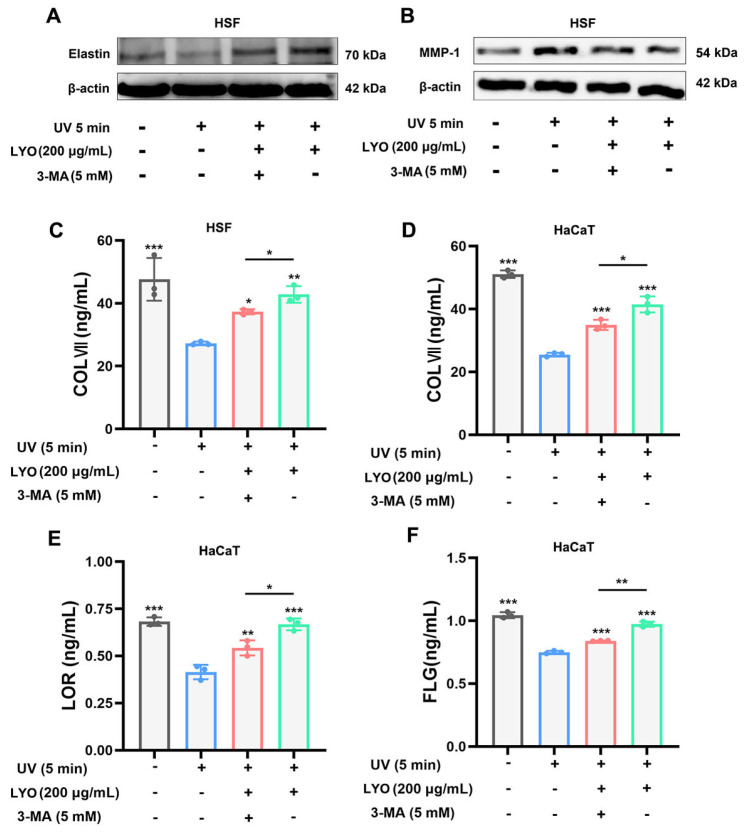
LYO exerts anti-aging effects by mediating autophagy. HSF cells were treated with 5 mM 3-MA with or without 200 μg/mL of LYO for 12 h. The expression of (**A**) elastin and (**B**) MMP-1 was determined by Western blotting. (**C**) The expression of COL VII was analyzed by ELISA, *n* = 3. HaCaT cells were treated with 5 mM 3-MA with or without 200 μg/mL of LYO for 12 h. (**D**–**F**) The expression of COL VII, LOR, and FLG were analyzed by ELISA, *n* = 3. Data are presented as mean ± SD. * *p* < 0.05, ** *p* < 0.01, *** *p* < 0.001, vs. the indicated groups.

Autophagy is a core biological process that maintains cellular homeostasis and resists senescence. The present study showed that LYO exerts a protective effect against UVB-induced photoaging in skin cells. To investigate whether its mechanism depends on the activation of the autophagic pathway, additional experiments were performed using the autophagy inhibitor 3-MA to evaluate the correlation between autophagy activation and anti-aging effects. As shown in Figure 5A–B, compared with the control group, UVB irradiation significantly downregulated the expression of elastin in HSF cells while markedly upregulating the expression of MMP-1, a key enzyme involved in collagen degradation, which is consistent with typical photoaging characteristics. Pretreatment with LYO significantly reversed the UVB-induced damage, upregulating elastin expression and downregulating MMP-1 expression, thereby exerting an anti-photoaging effect. After the addition of the autophagy inhibitor 3-MA, the upregulation of elastin and the downregulation of MMP-1 by LYO were significantly attenuated, suggesting that these effects of LYO rely on autophagy pathway activation (Figures S15 and S16). Furthermore, UVB irradiation markedly reduced the expression of COL VII in HSF cells, whereas LYO pretreatment significantly restored COL VII levels. However, treatment with 3-MA significantly blocked this protective effect of LYO, resulting in a decrease in COL VII compared with the LYO-alone group (Figure 5C). In HaCaT cells, UVB significantly inhibited COL VII expression, and 3-MA abolished the protective effect of LYO (Figure 5D). LOR and FLG are essential structural proteins that maintain epidermal barrier function, and UVB damage reduces their expression, leading to impaired skin barrier. Accordingly, UVB irradiation significantly decreased the secretion levels of both LOR and FLG. LYO pretreatment markedly upregulated the secretion of these two proteins and restored epidermal barrier integrity. Furthermore, 3-MA significantly suppressed this upregulating effect of LYO, resulting in markedly lower levels of LOR and FLG compared with the LYO-alone group (Figure 5E–F). Collectively, the autophagy inhibitor 3-MA significantly reversed the protective effects of LYO on the abovementioned photoaging-related indicators, confirming that autophagy activation is a key mechanism underlying the anti-skin photoaging effect of LYO.

## 4. Discussion

Skin photoaging refers to structural and functional abnormalities of the skin induced by UVB exposure, which severely compromises skin health. In the present study, a yeast strain *Sporidiobolus pararoseus* was isolated from the roots of red camellia, and a novel biotransformation product, designated LYO, was obtained via co-fermentation with *Camellia japonica* seed oil. GC-MS analysis revealed that the main methylated derivatives of LYO were methyl oleate (71.01%), methyl linoleate (11.95%), methyl palmitate (9.82%), and methyl stearate (3.39%). Among these, monounsaturated oleic acid accounted for more than 70%, which was largely consistent with the fatty acid profile of conventional *Camellia japonica* seed oil. Notably, small amounts of conjugated linoleic acid derivatives, such as methyl elaidate (2.71%), were detected in LYO, suggesting that the fermentation process may trigger the isomerization or bioconversion of certain fatty acid, thereby conferring potential photoprotective activities [44].

With respect to biosafety profiles, CCK-8 assays revealed that LYO exerted no cytotoxic effects on either HSF or HaCaT cells within the concentration range of 12.5~400 μg/mL. Furthermore, LYO significantly stimulated the proliferation of HaCaT cells at 100~400 μg/mL. The favorable biocompatibility of LYO thereby establishes a solid basis for its further development and practical application in skincare preparations. Autophagy plays a complex regulatory role in the skin’s stress response to photoaging. UVB radiation elevates ROS levels, intensifying damage to proteins, lipids, and DNA within cells. As an adaptive response, autophagy helps mitigate ROS levels by alleviating nuclear damage, repairing DNA, and clearing damaged organelles, thereby delaying the onset of photoaging [45]. RT-qPCR analysis confirmed that LYO significantly upregulated the expression of the autophagic marker LC3B and downregulated p62 expression in both HaCaT and HSF cells, indicating that LYO effectively activates the autophagic pathway in skin cells. Further morphological observations and flow cytometric analyses revealed that LYO significantly alleviated UVB-induced cellular damage and reduced apoptotic rates. These findings are closely associated with the antioxidant and anti-apoptotic properties of LYO, and provide direct cellular evidence supporting its anti-photoaging effects.

At the molecular mechanistic level, UVB irradiation in HSF cells upregulated the expression of the senescence marker p21 and the collagen-degrading enzyme MMP-1 while downregulating the anti-aging protein SIRT1, the oxidative stress regulator FoxO3a, and the expression of collagens (COL I, COL III, COL IV, COL VII) and elastin. Treatment with LYO significantly reversed these alterations, suggesting that it not only inhibits collagen degradation but also promotes the synthesis of extracellular matrix components. These observations indicate that LYO may interfere with this pathological process via modulation of the autophagy lysosome pathway. In HaCaT cells, LYO not only reversed the UVB-induced upregulation of p21 and downregulation of SIRT1 and FoxO3a, but also significantly enhanced the expression of skin barrier-related proteins, including LOR, FLG, and CER. These proteins play pivotal roles in maintaining the structural integrity of the stratum corneum, preserving skin hydration, and defending against external insults. The stimulatory effects of LYO on these barrier proteins imply that it may achieve comprehensive anti-photoaging efficacy through an autophagy–barrier axis.

In the present study, intervention experiments with 3-MA further verified the central role of autophagy in the anti-photoaging effects of LYO. As a well-established class III PI3K inhibitor, 3-MA blocks the early stage of autophagosome formation [46]. Our results demonstrated that pretreatment with 3-MA markedly attenuated the regulatory effects of LYO on the upregulation of elastin and downregulation of MMP-1 in HSF cells, and also partially abolished the stimulatory effects of LYO on the expression of COL VII, LOR, and FLG in HaCaT cells. Collectively, these findings strongly indicate that the anti-photoaging activity of LYO is dependent on activation of the autophagic pathway.

This work focuses on the photoprotective properties of fermented *Camellia japonica* seed oil. Nevertheless, while this study provides new insights into the anti-aging mechanisms of *Camellia japonica* seed oil, several future research directions should be noted. First, the current study relies mainly on HSF and HaCaT cell lines, which, despite their reproducibility, fail to fully recapitulate the intricate physiological microenvironment of human skin. Further validation using three-dimensional human skin equivalents or in vivo animal models is therefore indispensable for evaluating the clinical translational potential of LYO. Second, although the causal role of autophagy in LYO’s protective effects has been preliminarily confirmed using the pharmacological inhibitor 3-MA, the upstream regulatory network, particularly, whether LYO directly modulates the mTOR or AMPK pathways, remains to be elucidated. Third, given the multifactorial nature of skin aging, exploring synergistic combinations of LYO with other natural products or active ingredients could provide a theoretical basis for development of efficient combination of anti-aging therapies.

## 5. Conclusions

In summary, this study reports for the first time a novel *Camellia japonica* seed oil derivative, LYO, prepared via microbial fermentation, and systematically validates its protective effects against UVB-induced photoaging damage in skin cells through the activation of the autophagic pathway. LYO not only suppresses cellular senescence, apoptosis, and extracellular matrix degradation, but also enhances the expression of skin barrier-related proteins, thereby exerting multi-target and multi-level anti-photoaging activities. These findings provide theoretical and experimental foundations for the development of anti-skin photoaging strategies based on natural fermented products, and also open new avenues for the high-value utilization of *Camellia japonica* seed oil.

## Figures and Tables

**Table 1:** Chemical composition of LYO analyzed by GC-MS.

Peak	Rt (min)	Component	Content (mg/g)	Percentage (%)
1	17.210	Methyl myristate	0.1506	0.071
2	22.601	Methyl palmitoleate	0.3975	0.187
3	23.247	Methyl palmitate	20.9013	9.815
4	25.710	Methyl cis-10-heptadecenoate	0.2290	0.108
5	26.456	Methyl heptadecanoate	0.1494	0.070
6	28.815	Methyl linoleate	25.4378	11.945
7	29.177	Methyl oleate	151.2109	71.005
8	29.258	Methyl elaidate	5.7788	2.714
9	29.927	Methyl stearate	7.2248	3.393
10	38.374	Methyl eicosenoate	1.1318	0.531
11	58.204	Methyl (15Z)-15-tetracosenoate	0.1608	0.076
12	58.726	Methyl tetracosanoate	0.1861	0.087

## Data Availability

The original contributions presented in this study are included in the article; further inquiries can be directed at the corresponding author.

## Supplementary Materials

The following supporting information can be downloaded at: https://www.mdpi.com/article/10.3390/life16071145/s1, Figure S1: GC-MS analysis of LYO components; Figure S2: Autophagy-associated morphological changes in HSF and HaCaT cells following treatment. Representative images of HSF (scale bar: 200 μm) and HaCaT (Scale bar: 20 μm). Red arrows indicate cytoplasmic vacuolization, a morphological hallmark of autophagic activation; Figure S3: Corresponding quantitative analysis of p21 in HSF cells (*n* = 3). Data are presented as mean ± SD. * *p* < 0.05, ** *p* < 0.01, *** *p* < 0.001; Figure S4: Corresponding quantitative analysis of SIRT 1 in HSF cells (*n* = 3). Data are presented as mean ± SD. * *p* < 0.05, ** *p* < 0.01, *** *p* < 0.001; Figure S5: Corresponding quantitative analysis of FoxO3a in HSF cells (*n* = 3). Data are presented as mean ± SD. * *p* < 0.05, ** *p* < 0.01, *** *p* < 0.001; Figure S6: Corresponding quantitative analysis of Elastin in HSF cells (*n* = 3). Data are presented as mean ± SD. * *p* < 0.05, ** *p* < 0.01, *** *p* < 0.001; Figure S7: Corresponding quantitative analysis of latent MMP-1 in HSF cells (*n* = 3). Data are presented as mean ± SD. * *p* < 0.05, ** *p* < 0.01, *** *p* < 0.001; Figure S8: Corresponding quantitative analysis of COL I in HSF cells (*n* = 3). Data are presented as mean ± SD. * *p* < 0.05, ** *p* < 0.01, *** *p* < 0.001; Figure S9: Corresponding quantitative analysis of COL III in HSF cells (*n* = 3). Data are presented as mean ± SD. * *p* < 0.05, ** *p* < 0.01, *** *p* < 0.001; Figure S10: Corresponding quantitative analysis of COL IV in HSF cells (*n* = 3). Data are presented as mean ± SD. * *p* < 0.05, ** *p* < 0.01, *** *p* < 0.001; Figure S11: Corresponding quantitative analysis of p21 in HaCaT cells (*n* = 3). Data are presented as mean ± SD. * *p* < 0.05, ** *p* < 0.01, *** *p* < 0.001; Figure S12: Corresponding quantitative analysis of FoxO3a in HaCaT cells (*n* = 3). Data are presented as mean ± SD. * *p* < 0.05, ** *p* < 0.01, *** *p* < 0.001; Figure S13: Corresponding quantitative analysis of SIRT 1 in HaCaT cells (*n* = 3). Data are presented as mean ± SD. * *p* < 0.05, ** *p* < 0.01, *** *p* < 0.001; Figure S14: Corresponding quantitative analysis of COL IV in HaCaT cells (*n* = 3). Data are presented as mean ± SD. * *p* < 0.05, ** *p* < 0.01, *** *p* < 0.001; Figures S15: HSF cells were treated with 5 mM 3-MA with or without 200 μg/mL of LYO for 12 h. Corresponding quantitative analysis of Elastin in HSF cells (*n* = 3). Data are presented as mean ± SD. * *p* < 0.05, ** *p* < 0.01, *** *p* < 0.001; Figures S16: HSF cells were treated with 5 mM 3-MA with or without 200 μg/mL of LYO for 12 h. Corresponding quantitative analysis of latent MMP-1 in HSF cells (*n* = 3). Data are presented as mean ± SD. * *p* < 0.05, ** *p* < 0.01, *** *p* < 0.001.
