# Supplementary material for: Camellia japonica Seed Oil Fermented by Sporidiobolus pararoseus Prevents Skin Cellular Photoaging by Inducing Autophagy
Source: Life (Basel). 2026 Jul 10;16(7):1145. doi: 10.3390/life16071145 (PMC13412233; doi:10.3390/life16071145)
Supplement: Supplementary file 1 [file life-16-01145-s001.zip › life-4340303-supplementary.pdf]

Supporting Information

Camellia japonica **Seed Oil Fermented by** ***Sporidiobolus pararoseus*** **Prevents Skin Cellular Photoaging by Inducing Autophagy**

Bai Lv[1#], Jiale Meng[1#], Xichao Zhang[1], Guangtao Li[2,3], Hongqi Gao[2,3], Yi Jiang[2,3], Zhanwei Zhou[4*] and Gang Chen[1*]

[1] *School of Pharmacy, Qingdao University, Qingdao 266071, China*

[2] *Anti-Cellular Senescence Joint Research Laboratory of Forest Cabin & Shanghai Jiao Tong University, Shanghai 201600 China*

[3] *Shanghai Forest Cabin Cosmetics Group Co., Ltd. Shanghai 201600, China*

[4] *Department of Pharmaceutics, China Pharmaceutical University, Nanjing 210009, China*

[#] *Bai Lv and Jiale Meng contributed equally to this work.*

[*] *Correspondence:*

Gang Chen: chengang@uhrs.edu.cn

Zhanwei Zhou: zwzhou@cpu.edu.cn

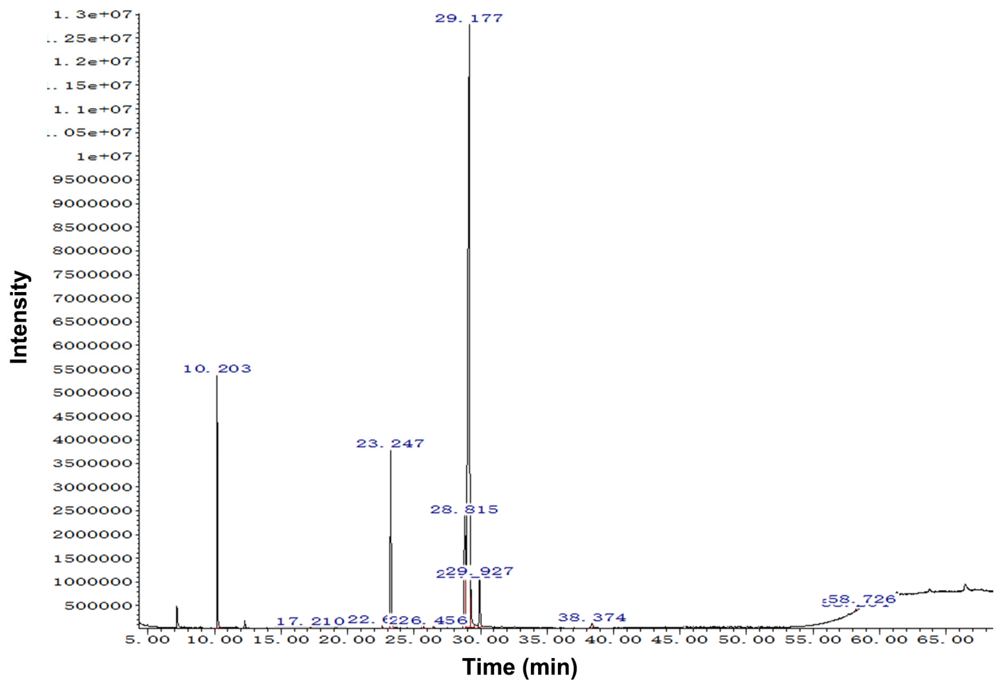

Figure S1. GC-MS analysis of LYO components.

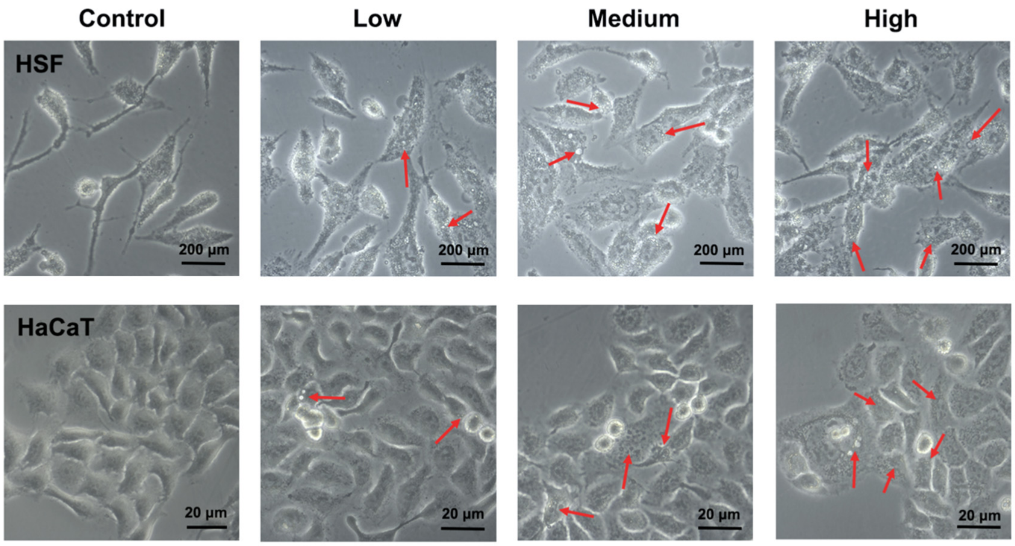

Figure S2. Autophagy-associated morphological changes in HSF and HaCaT cells following treatment. Representative images of HSF (scale bar: 200 μm) and HaCaT (Scale bar: 20 μm). Red arrows indicate cytoplasmic vacuolization, a morphological hallmark of autophagic activation.

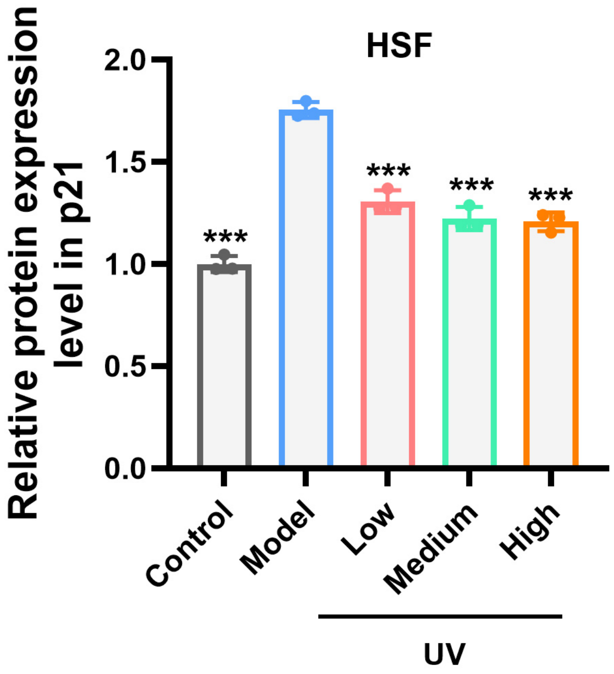

Figure S3. Corresponding quantitative analysis of p21 in HSF cells (n = 3). Data are presented as mean ± SD. $^{*}P < 0.05$, $^{**}P < 0.01$, $^{***}P < 0.001$.

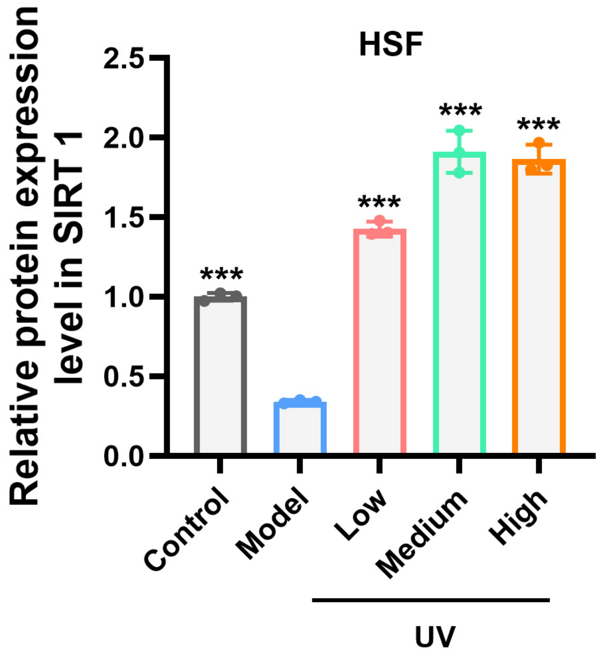

Figure S4. Corresponding quantitative analysis of SIRT 1 in HSF cells (n = 3). Data are presented as mean ± SD. $^{*}P < 0.05$, $^{**}P < 0.01$, $^{***}P < 0.001$.

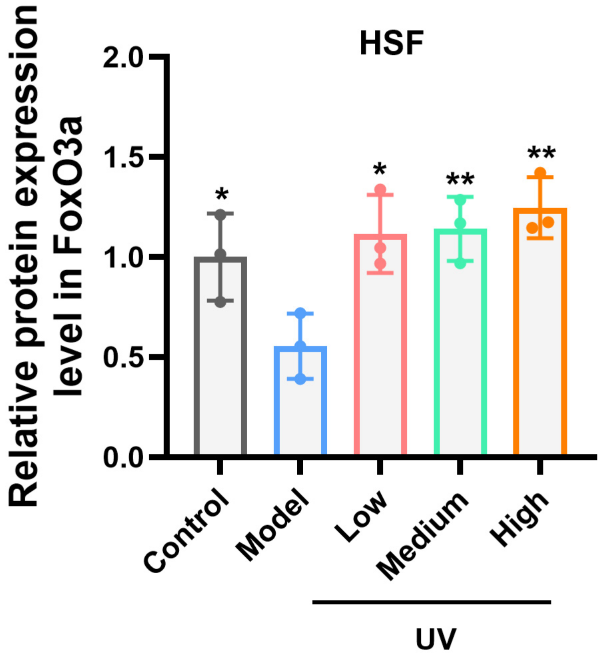

Figure S5. Corresponding quantitative analysis of FoxO3a in HSF cells (n = 3). Data are presented as mean ± SD. $^{*}P < 0.05$, $^{**}P < 0.01$, $^{***}P < 0.001$.

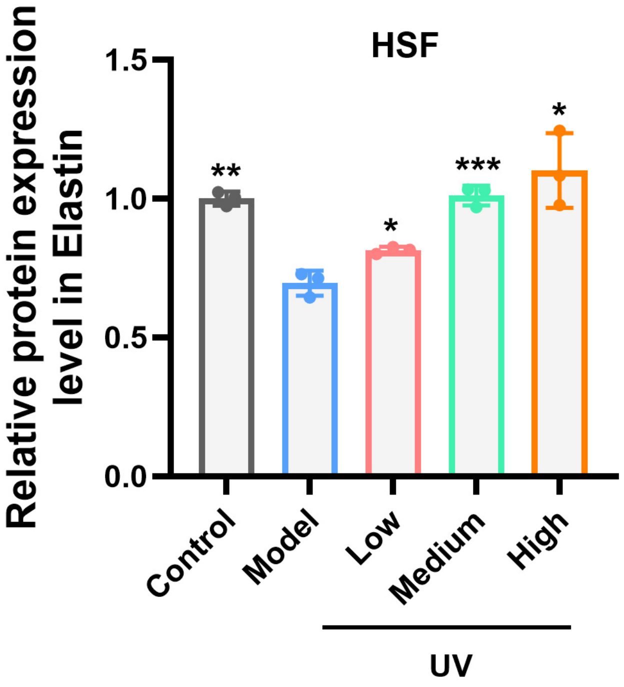

Figure S6. Corresponding quantitative analysis of Elastin in HSF cells (n = 3). Data are presented as mean ± SD. $^{*}P < 0.05$, $^{**}P < 0.01$, $^{***}P < 0.001$.

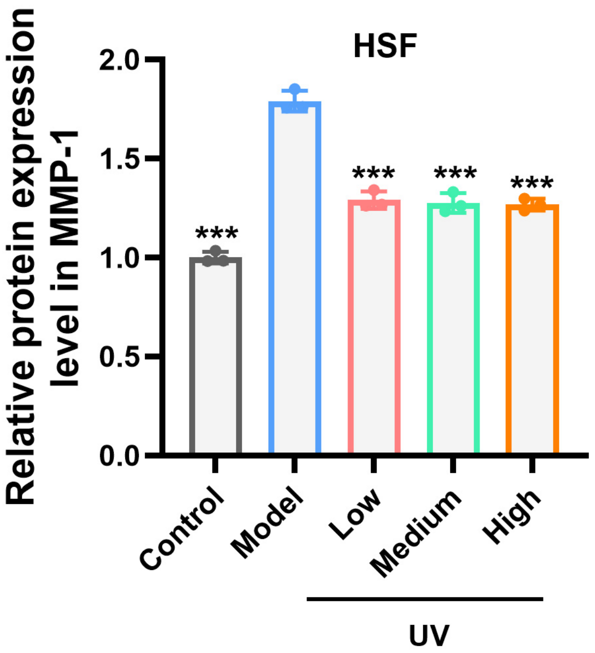

Figure S7. Corresponding quantitative analysis of MMP-1 in HSF cells (n = 3). Data are presented as mean ± SD. $^{*}P < 0.05$, $^{**}P < 0.01$, $^{***}P < 0.001$.

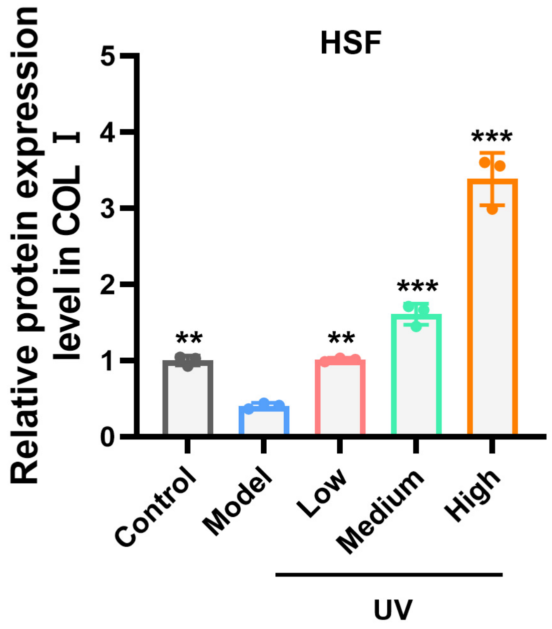

Figure S8. Corresponding quantitative analysis of COL I in HSF cells (n = 3). Data are presented as mean ± SD. $^{*}P < 0.05$, $^{**}P < 0.01$, $^{***}P < 0.001$.

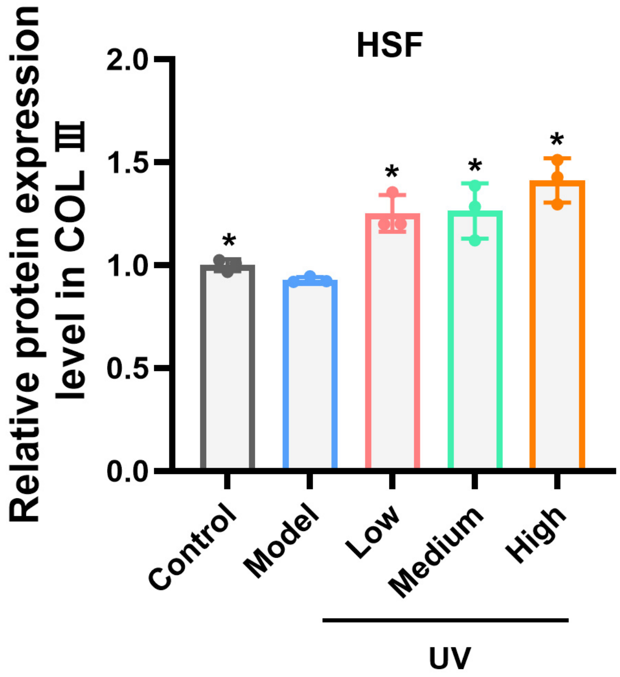

Figure S9. Corresponding quantitative analysis of COL III in HSF cells (n = 3). Data are presented as mean ± SD. $^{*}P < 0.05$, $^{**}P < 0.01$, $^{***}P < 0.001$.

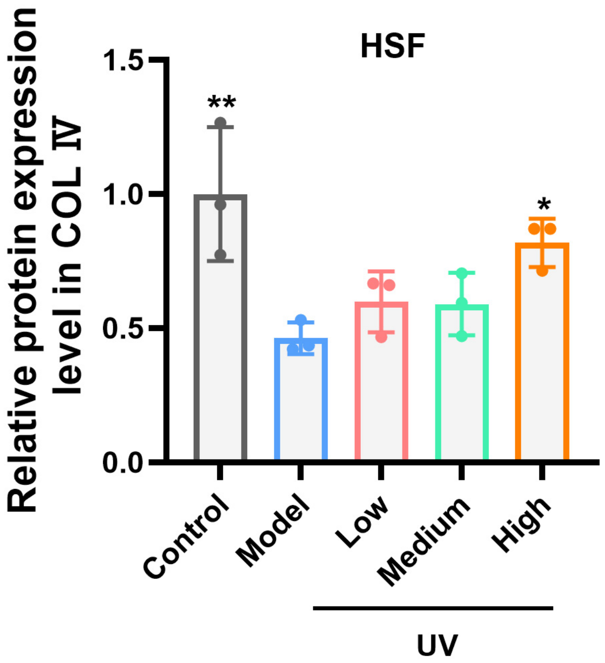

Figure S10. Corresponding quantitative analysis of COL IV in HSF cells (n = 3). Data are presented as mean ± SD. $^{*}P < 0.05$, $^{**}P < 0.01$, $^{***}P < 0.001$.

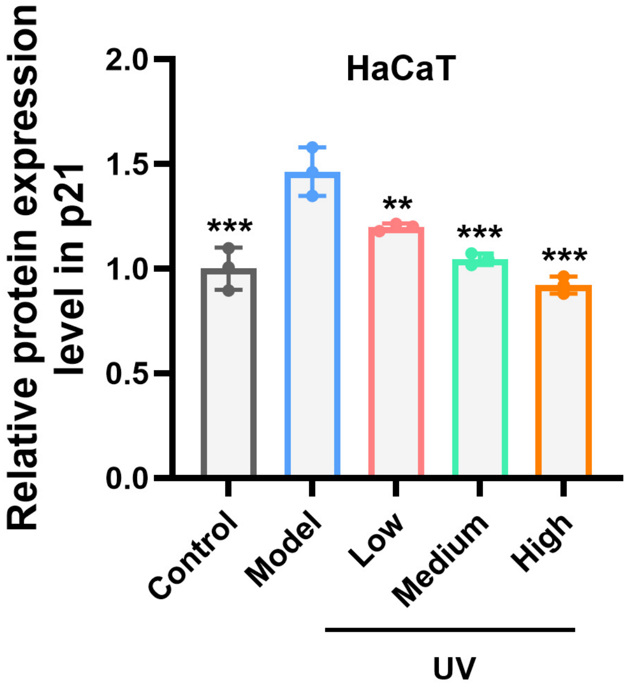

Figure S11. Corresponding quantitative analysis of p21 in HaCaT cells (n = 3). Data are presented as mean ± SD. $^{*}P < 0.05$, $^{**}P < 0.01$, $^{***}P < 0.001$.

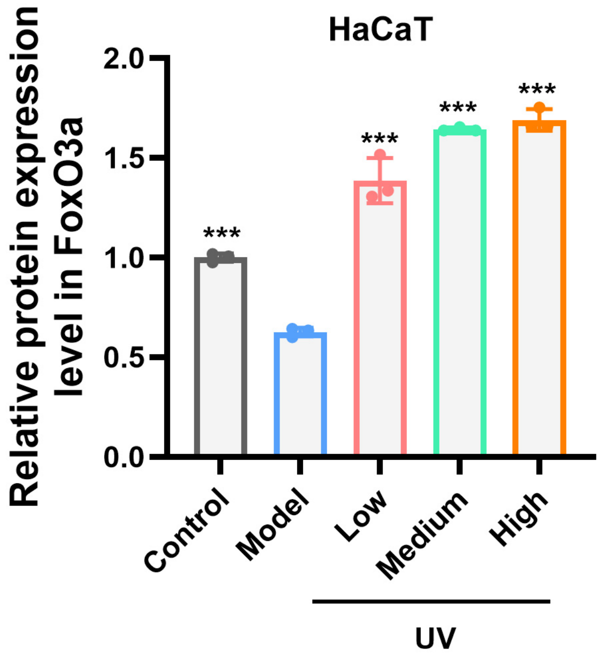

Figure S12. Corresponding quantitative analysis of FoxO3a in HaCaT cells (n = 3). Data are presented as mean ± SD. $^{*}P < 0.05$, $^{**}P < 0.01$, $^{***}P < 0.001$.

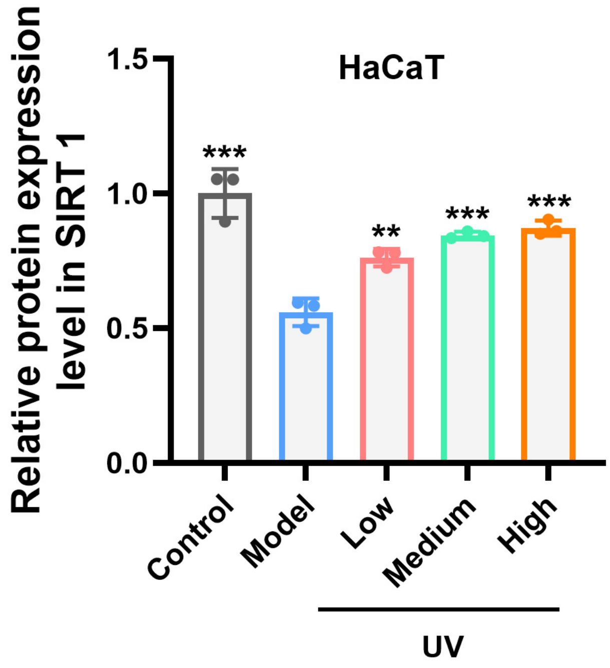

Figure S13. Corresponding quantitative analysis of SIRT 1 in HaCaT cells (n = 3). Data are presented as mean ± SD. $^{*}P < 0.05$, $^{**}P < 0.01$, $^{***}P < 0.001$.

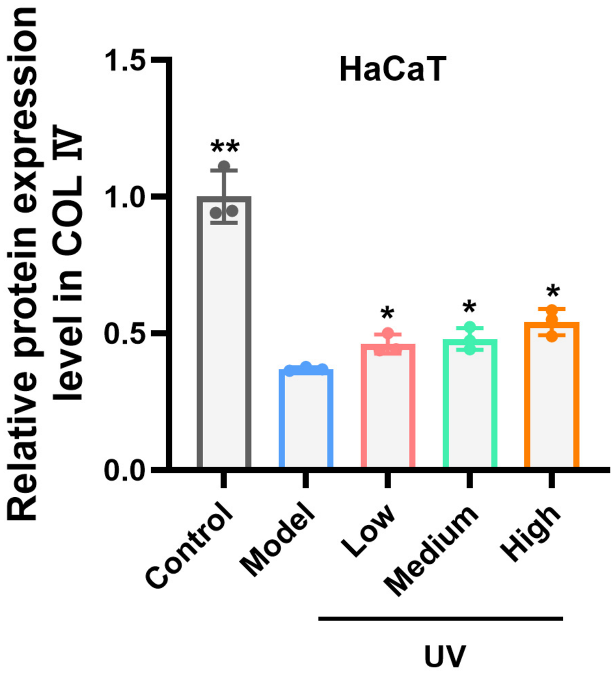

Figure S14. Corresponding quantitative analysis of COL IV in HaCaT cells (n = 3). Data are presented as mean ± SD. $^{*}P < 0.05$, $^{**}P < 0.01$, $^{***}P < 0.001$.

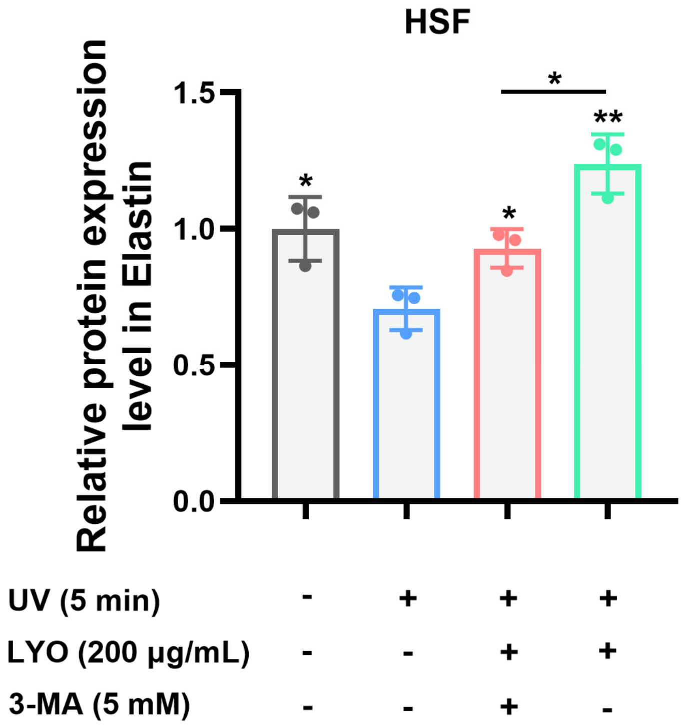

Figures S15. HSF cells were treated with 5 mM 3-MA with or without 200 µg/mL of LYO for 12 h. Corresponding quantitative analysis of Elastin in HSF cells (n = 3). Data are presented as mean ± SD. $^{*}P < 0.05$, $^{**}P < 0.01$, $^{***}P < 0.001$.

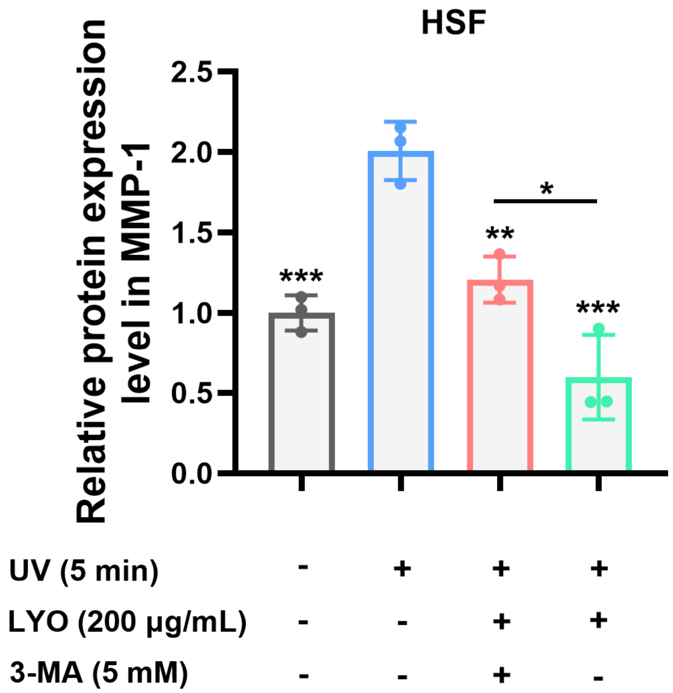

Figures S16. HSF cells were treated with 5 mM 3-MA with or without 200 μg/mL of LYO for 12 h. Corresponding quantitative analysis of MMP-1 in HSF cells (n = 3). Data are presented as mean ± SD. $^{*}P < 0.05$, $^{**}P < 0.01$, $^{***}P < 0.001$.
